# PspA Family Distribution, Antimicrobial Resistance and Serotype of *Streptococcus pneumoniae* Isolated from Upper Respiratory Tract Infections in Japan

**DOI:** 10.1371/journal.pone.0058124

**Published:** 2013-03-06

**Authors:** Muneki Hotomi, Akihisa Togawa, Masamitsu Kono, Yorihiko Ikeda, Shin Takei, Susan K. Hollingshead, David E. Briles, Kenji Suzuki, Noboru Yamanaka

**Affiliations:** 1 Department of Otolaryngology-Head and Neck Surgery, Wakayama Medical University, Wakayama-shi, Wakayama, Japan; 2 Department of Microbiology, University of Birmingham at Alabama, Birmingham, Alabama, United States of America; 3 Department of Otolaryngology, Second Hospital, School of Medicine, Fujita Health University, Toyoake, Aichi, Japan; 4 The Surveillance Subcommittee, The Japan Society for Infectious Diseases in Otolaryngology, Nagoya, Aichi, Japan; Glaxo Smith Kline, Denmark

## Abstract

**Background:**

The protection against pneumococcal infections provided by currently available pneumococcal polysaccharide conjugate vaccines are restricted to the limited number of the serotypes included in the vaccine. In the present study, we evaluated the distribution of the pneumococcal capsular type and surface protein A (PspA) family of pneumococcal isolates from upper respiratory tract infections in Japan.

**Methods:**

A total of 251 *S. pneumoniae* isolates from patients seeking treatment for upper respiratory tract infections were characterized for PspA family, antibiotic resistance and capsular type.

**Results:**

Among the 251 pneumococci studied, the majority (49.4%) was identified as belonging to PspA family 2, while most of the remaining isolates (44.6%) belonged to family 1. There were no significant differences between the distributions of PspA1 versus PspA2 isolates based on the age or gender of the patient, source of the isolates or the isolates’ susceptibilities to penicillin G. In contrast, the frequency of the *mefA* gene presence and of serotypes 15B and 19F were statistically more common among PspA2 strains.

**Conclusion:**

The vast majority of pneumococci isolated from the middle ear fluids, nasal discharges/sinus aspirates or pharyngeal secretions represented PspA families 1 and 2. Capsular serotypes were generally not exclusively associated with certain PspA families, although some capsular types showed a much higher proportion of either PspA1 or PspA2. A PspA-containing vaccine would potentially provide high coverage against pneumococcal infectious diseases because it would be cross-protective versus invasive disease with the majority of pneumococci infecting children and adults.

## Introduction

*Streptococcus pneumoniae* (*S. pneumoniae*) is a major etiological agent causing various infectious diseases ranging from non-invasive diseases such as acute otitis media (AOM), rhinosinusitis and pneumonia to invasive disease such as sepsis and meningitis in young children and the elderly [1]–[4]. In recent decades, penicillin resistant *S. pneumoniae* (PRSP) have evolved at a rapid pace into a global problem [5]–[7]. The high prevalence of antimicrobial resistant pneumococci has further emphasized the importance of pneumococcal vaccines [8], [9].

Currently available pneumococcal vaccines are based on capsular polysaccharides. Although the 23-valent polysaccharide vaccine (23 PPV) is immunogenic and protective in most adults, it has been shown to be poorly efficacious in children younger than 2 years of age [10]. In contrast, the 7-valent pneumococcal conjugate vaccine (7 PCV) is highly efficacious at preventing bacteremic disease in children under 5 years of age [11]–[14]. Promising results regarding the prevention of pneumonia and AOM, reducing nasopharyngeal carriage of vaccine serotypes, and elicitation of herd immunity against vaccine serotypes have also been reported for PCV [15]. However, the protection is restricted to the limited number of the serotypes included in the vaccine. In recent years the protection afforded by the conjugate vaccine has begun to be eroded by an increasing frequency of infections with pneumococcal strains not covered by the vaccine [16], [17]. An ideal pneumococcal vaccine would be immunogenic in all young children, the age group for whom pneumococcal infection and mortality is the highest in the developing world. An ideal vaccine would also protect against pneumococci regardless of their capsular types [18].

Pneumococcal surface protein A (PspA) is an important virulent factor expressed by all pneumococci that is essential for full virulence in invasive disease, and contributes to colonization [19]–[21]. It is highly immunogenic and protective against invasive disease as well as nasal colonization in mice. Protective antibody to PspA is elicited in the alpha helical and proline-rich domains of PspA. Of the ∼300 amino acid alpha-helical domain protective, its 100 C-terminal amino acids, known as the clade/family-defining region are responsible for much of the elicited protection [22]–[25]. However, the N-terminal 100 amino acids are also protection-eliciting [24], [25]. All three protection-eliciting regions exhibit variability in their mosque sequence but contain many shared sequences some of which are highly conserved [22], [26]. These shared sequences outside the family-defining regions and shared sequence between families within the family-defining region explain why immunity to PspAs of one family can often elicit some or complete protection against strains expressing PspAs of other families. Since virtually all pneumococci have at least slightly different PspA sequences, we regard virtually all protection by antibody to PspA as cross-protection. Immunity to PspA is highly cross-protective against invasive disease [27], [28]. Thus, a vaccine containing at least three different PspAs should be able to provide redundant protection against all pneumococci. Both intranasal and humoral immunization with PspA can also protect against colonization [29]–[31]. Consequently, PspA is an attractive candidate antigen for the development of new effective vaccines [32].

Since capsular type distribution is not uniform world wide, it is important to know the overall distribution of the PspA family expressed in pneumococcal strains at multiple sites around the world to make sure the PspA molecules represented in a vaccine will be effective world wide [33]–[35]. In the present study, we evaluated the distribution of PspA family types among pneumococcal isolates from upper respiratory tract infections in Japan.

## Materials and Methods

### *S. pneumoniae* Strains

Between January and May 2003, the Japanese Society of Infectious Disease in Otorhinolaryngology conducted the fourth nationwide surveillance of the bacterial pathogens responsible for otorhinolaryngological infections. A total of 251 *S. pneumoniae* isolates were collected from 251 patients treated for the upper respiratory tract infections including AOM, rhinosinusitis and pharyngotonsillitis during these periods. All pneumococcal strains were identified by alpha-hemolysis and colony morphology on 5% sheep blood agar, Gram’s stained smear, optochin disk sensitivity, bile solubility, and the presence of *ply* gene by polymerase chain reaction (PCR). The patients ranged in age from 0 to 68 years old, with 125 females and 126 males. Among the isolates, 57 (22.7%) were from the middle ear fluids (MEFs), 88 (35.1%) were from nasal discharges or sinus aspirates and 106 (42.2%) were from the pharyngeal secretions (Table 1).

**Table 1:** Distribution of *S. pneumoniae* serotypes based on their susceptibilities to PCG.

Category	Sub-category	Total	Susceptibility to PCG (µg/ml)			*p*-value
			PSSP	PISP	PRSP	DRSP v.s. PSSP
Gender	Female	125 (49.8%)	50 (19.9%)	48 (19.1%)	27 (10.8%)	*p* = 0.362
	Male	126 (50.2%)	43 (17.1%)	56 (22.3%)	27 (10.8%)	
Age	0–2	92 (36.7%)	16 (6.4%)	50 (19.9%)	26 (10.4%)	*p*<0.001*
	3–5	37 (14.7%)	16 (6.4%)	17 (6.8%)	4 (1.6%)	
	6–12	25 (10%)	18 (7.2%)	7 (2.8%)	0 (0%)	
	13–20	7 (2.8%)	4 (1.6%)	2 (0.8%)	1 (0.4%)	
	21–50	72 (28.7%)	30 (12.0%)	22 (8.8%)	20 (8.0%)	
	≥51	18 (7.2%)	9 (3.6%)	6 (2.4%)	3 (1.2%)	
Origin	Middle ear fluids	57 (22.7%)	21 (8.4%)	27 (10.8%)	9 (3.6%)	*p* = 1.000
	Nasal discharge/Sinus aspirates	88 (35.1%)	37 (14.7%)	34 (13.5%)	17 (6.8%)	*p* = 0.273
	Pharyngeal secretions	106 (42.2%)	35 (13.9%)	43 (17.1%)	28 (11.2%)	*p* = 0.291
Serotype	1	2 (0.8%)	1 (0.4%)	0 (0%)	1 (0.4%)	*p* = 1.000
	3	14 (5.6%)	13 (5.2%)	1 (0.4%)	0 (0%)	*p*<0.001
	4	1 (0.4%)	0 (0%)	0 (0%)	1 (0.4%)	*p* = 1.000
	6A	16 (6.4%)	6 (2.4%)	6 (2.4%)	4 (1.6%)	*p* = 1.000
	6B	37 (14.7%)	14 (5.6%)	17 (6.8%)	6 (2.4%)	*p* = 1.000
	9V	5 (2.0%)	3 (1.2%)	2 (0.8%)	0 (0%)	*p* = 0.667
	14	20 (8.0%)	2 (0.8%)	13 (5.2%)	5 (2.0%)	*p* = 0.002
	15B	7 (2.8%)	4 (1.6%)	3 (1.2%)	0 (0%)	*p* = 0.429
	19A	5 (2.0%)	4 (1.6%)	1 (0.4%)	0 (0%)	*p* = 0.064
	19F	52 (20.7%)	8 (3.2%)	25 (10.0%)	19 (7.6%)	*p*<0.001
	23F	41 (16.3%)	11 (4.4%)	18 (7.2%)	12 (4.8%)	*p* = 0.029
	G23	6 (2.4%)	4 (1.6%)	2 (0.8%)	0 (0%)	*p* = 0.198
	Others	45 (17.9%)	23 (9.2%)	16 (6.4%)	6 (2.4%)	*p* = 0.040
Total		251 (100%)	93 (37.1%)	104 (41.4%)	54 (21.5%)	

G23: serogroup 23 strains except serotype 23F. PCG: penicillin G. PSSP: penicillin susceptible *S. pneumoniae*. PISP: penicillin intermediately resistant *S. pneumoniae*. PRSP: penicillin resistant *S. pneumoniae*. DRSP: PRSP+PISP. Others: serotypes not included in 23 PPV.

* comparison between ≤2 y.o. vs. ≥3 y.o.

Susceptibility to penicillin G (PCG) was tested by a broth dilution standard method according to the guidelines of the Clinical and Laboratory Standards Institute (CLSI). The CLSI published revised susceptibility breakpoints for penicillin and *S. pneumoniae* in 2008. The revised susceptibility breakpoint is ≤2 µg/ml for non-meningeal infections treated with parental penicillin. In this study, categorization of penicillin susceptibility according to the former CLSI guidelines was applied because most of the cases were treated with oral penicillin. Strains with MICs of PCG ≥2 µg/ml were interpreted as penicillin resistant *S. pneumoniae* (PRSP), strains with MICs from 0.1 to 1 µg/ml were classified as penicillin intermediate resistant *S. pneumoniae* (PISP), and strains with MICs ≤0.06 µg/ml were interpreted as penicillin susceptible *S. pneumoniae* (PSSP). During the assay, *S. pneumoniae* strains ATCC 49619 and ATCC BAA-334 were used as susceptible controls for quality assurance [36].

### Serotype

All isolates were serotyped or serogrouped by the capsular quellung reaction method with pneumococcal capsule specific antisera (Statens Serum Institute, Copenhagen, Denmark), as recommended by the manufacturer. Strains of serotypes 4 (ATCC BAA-334) and 19F (ATCC 49619) obtained from the American Type Culture Collection 169 (ATCC, Manassas, VA, USA) were used for quality control in every reaction.

### PspA Family Classification

PspAs were classified into three families by PCR. Briefly, genomic DNA was extracted from pneumococcal isolates as described and stored at 4°C [37]. PCR were carried out in a standard PCR mixture (QIAGEN, Valencia, CA, USA) of 25 µl containing 2.5 mM MgCl_2_, 200 µM dNTPs (each), 50 pmol of primers, and 2.5 U of *Taq* DNA polymerase. The oligonucleotide primers (LSM12, SKH63, SKH52, SKH41, SKH42, SKH02, ply1, and ply2) reported by Hollingshead et al were used in this study [37]. Primers for PspA family 1 (PspA1) and PspA family 2 (PspA2) were LSM12/SKH63 and LSM12/SKH52, respectively. Primers for PspA family 3 (PspA3) were SKH41 and SKH 42. Primers LSM12 and SKH02 were used for testing the presence of *pspA* gene. Primers ply1 and ply2 were used for testing the presence of the pneumolysin gene.

The PCR conditions were 95°C for 3 min; then 30 cycles of 95°C for 1 min, 62°C for 1 min and 72°C for 3 min, and finally 72°C for 10 min. The optimal annealing temperature was 62°C. The isolates that were not initially amplified were further processed with the same cycling pattern at an annealing temperature of 58°C, or, if that also failed, of 55°C. Isolate that were not typed after the lower annealing temperatures in the family 1, 2, and 3 tests were classified as nontypeable PspA (PspA NT). An additional two tests were used to verify that the PspA NT isolates were truly pneumococcal isolates. One test was for the presence of the pneumolysin gene and another test was for the presence of the *pspA* gene. A single isolate that was amplified by the ply primers and not amplified by any of the PspA primers was classified as PspA null.

Three microliters of the PCR products were loaded on 0.8% agarose gels, electrophoresed at 80 V for 1 h, and stained with 0.5 µg/ml ethidium bromide.

### Statistical Analysis

All data were statistically analyzed by using Prism 4 (GraphPad Software, Inc., La Jolla, CA, USA). A two tailed chi-square test or Fisher’s exact test (for small group sizes) was used for categorical variables to test the significance of differences between groups. A *p*-value of <0.05 was considered statistically significant. The odds ratio (OR) and 95% confidential intervals (CIs) of individual serotypes were calculated relative to all other serotypes in the samples.

### Ethical Approval

The isolates used in this study are all clinical isolates obtained from patients with otorhinolaryngological infections as part of routine clinical diagnosis and management. The main ethical issue relates to specific consent for detailed characterization of an isolate from a clinical specimen taken from a patient on clinical ground. Because no information that would allow identification of the patients was collected in this study, this requirement was waived by the Institutional Review Board of the Ethical Committee of Wakayama Medical University. This study was therefore approved by the Institutional Review Board of the Ethical Committee of Wakayama Medical University.

## Results

### Distribution of Pneumococcal Serotypes Based on their Penicillin and Macrolide Susceptibilities

The distribution of *S. pneumoniae* serotypes based on their susceptibilities to PCG is listed in Table 1. Based on their susceptibility to PCG, the 251 pneumococcal isolates evaluated in this study were classified into three groups as follows: 93 (37.0%) PSSP, 104 (41.4%) PISP, and 54 (21.6%) PRSP. There were no significant differences in distributions of susceptibilities to PCG based on gender of the patients providing the strains, or based on the source of the isolates. Drug resistant *S. pneumoniae* (DRSP; PISP+PRSP) were frequently identified among children younger than 2 years old (OR 4.5, 95% CI 2.4–8.3, *p*<0.001).

The most common serotype was 19F (20.7%) followed by 23F (16.3%), 6B (14.7%), 14 (8.0%), 6A (6.4%) and 3 (5.6%). Among the serogroup 6 strains, we could not find the recently discovered serotype 6C and 6D strains. The distribution of *S. pneumoniae* serotypes based on their susceptibility to PCG was statistically significant (*p*<0.001). Serotype 3 (OR 25.5, 95% CI 3.3–198.6, *p*<0.001) was prevalent among the strains with MICs to PCG of ≤0.06 µg/ml. In contrast serotype 14 (OR 5.9, 95% CI 1.3–25.8, *p* = 0.002) and serotype 19F (OR 4.1, 95% CI 1.8–9.2, *p*<0.001) were frequently identified among DRSP strains. The isolated strains identified as serotypes 6A and 6B showed a broad spectrum of antibiotic resistance regardless of their susceptibility to PCG. The most common five serotypes (19F, 23F, 6B, 6A and 14) represented about 79.1% of the DRSP strains.

The distribution of *S. pneumoniae* serotypes based on their susceptibilities to macrolide is listed in Table 2. Based on the macrolide susceptibilities, the 251 isolates were classified into four groups as follows: 106 (42.2%) strains with the *ermB* gene, 75 (29.8%) strains with the *mefA* gene, 15 (6.0%) strains with both genes, and 55 (22.0%) strains without both genes. There were no significant differences in distributions of macrolide resistant traits based on the gender or based on the source of the isolates or age of the patients.

**Table 2:** Distribution of *S. pneumoniae* serotypes based on their macroride-resistant traits.

Category	Sub-category	Total	Macrolide resistance genes				*p*-value
			*ermB*	*mefA*	*ermB+mefA*	None	MLR v.s. MLS
Gender	Female	99 (39.4%)	51 (20.3%)	39 (15.5%)	9 (3.6%)	26 (10.4%)	*p* = 0.761
	Male	97 (38.6%)	55 (21.9%)	36 (14.3%)	6 (2.4%)	29 (11.6%)	
Age	0–2	76 (30.3%)	39 (15.5%)	32 (12.7%)	5 (2.0%)	16 (6.4%)	*p* = 0.208*
	3–5	28 (11.2%)	14 (5.6%)	12 (4.8%)	2 (0.8%)	9 (3.6%)	
	6–12	14 (5.6%)	11 (4.4%)	3 (1.2%)	0 (0%)	11 (4.4%)	
	13–20	4 (1.6%)	3 (1.2%)	0 (0%)	1 (0.4%)	3 (1.2%)	
	21–50	58 (23.1%)	29 (11.6%)	22 (8.8%)	7 (2.8%)	14 (5.6%)	
	≥51	16 (6.4%)	10 (4.0%)	6 (2.4%)	0 (0%)	2 (0.8%)	
Origin	Middle ear fluids	46 (18.3%)	34 (13.5%)	11 (4.4%)	1 (0.4%)	11 (4.4%)	*p* = 0.264
	Nasal discharge/Sinus aspirates	65 (25.9%)	35 (13.9%)	25 (10.0%)	5 (2.0%)	23 (9.2%)	*p* = 0.716
	Pharyngeal secretions	85 (33.9%)	37 (14.7%)	39 (15.5%)	9 (3.6%)	21 (8.4%)	*p* = 0.539
Serotype	1	1 (0.4%)	0 (0%)	1 (0.4%)	0 (0%)	1 (0.4%)	*p* = 0.391
	3	10 (4.0%)	9 (3.6%)	1 (0.4%)	0 (0%)	4 (1.6%)	*p* = 0.514
	4	1 (0.4%)	0 (0%)	1 (0.4%)	0 (0%)	0 (0%)	*p* = 0.515
	6A	13 (5.2%)	8 (3.2%)	4 (1.6%)	1 (0.4%)	3 (1.2%)	*p* = 1.000
	6B	27 (10.8%)	17 (6.8%)	7 (2.8%)	3 (1.2%)	10 (4.0%)	*p* = 0.397
	9V	4 (1.6%)	3 (1.2%)	0 (0%)	1 (0.4%)	1 (0.4%)	*p* = 1.000
	14	17 (6.8%)	10 (4.0%)	6 (2.4%)	1 (0.4%)	3 (1.2%)	*p* = 0.579
	15B	6 (2.4%)	5 (2.0%)	0 (0%)	1 (0.4%)	1 (0.4%)	*p* = 1.000
	19A	1 (0.4%)	1 (0.4%)	0 (0%)	0 (0%)	4 (1.6%)	*p* = 0.009
	19F	49 (19.5%)	15 (6.0%)	30 (12.0%)	4 (1.6%)	3 (1.2%)	*p* = 0.001
	23F	36 (14.3%)	19 (7.6%)	14 (5.6%)	3 (1.2%)	5 (2.0%)	*p*<0.001
	G23	5 (2.0%)	4 (1.6%)	1 (0.4%)	0 (0%)	1 (0.4%)	*p* = 0.147
	Others	26 (10.4%)	15 (6.0%)	10 (4.0%)	1 (0.4%)	19 (7.6%)	*p* = 1.000
Total		196 (78.1%)	106 (42.2%)	75 (29.9%)	15 (6.0%)	55 (21.9%)	*p* = 0.001

G23: serogroup 23 strains except serotype 23F. PCG: penicillin G. PSSP: penicillin susceptible *S. pneumoniae*. PISP: penicillin intermediately resistant *S. pneumoniae*. PRSP: penicillin resistant *S. pneumoniae*. DRSP: PRSP+PISP. Others: serotypes not included in 23 PPV.

* comparison between ≤2 y.o. vs. ≥3 y.o.

The distribution of *S. pneumoniae* serotypes based on their macrolide resistant traits is also statistically significant (*p* = 0.001). The *mefA* gene was most prevalent among isolates typed as serotype 19F (OR 4.7, 95% CI 2.5–8.9, *p*<0.001). Strains of the most predominant six serotypes (19F, 23F, 6B, 6A, 14, and 3) represented 60.6% of the total strains and about 77.6% of the strains with macrolide resistant genes.

### Distribution of PspA Families Based on their Serotypes and Penicillin Susceptibilities

Among the 251 pneumococci isolates studied, the 49.4% were identified as belonging to family 2 (PspA2), and 44.6% to family 1 (PspA1). Thus, 94.0% of the isolates included in this study were PspA1- or PspA2-positive isolates. Eight isolates (3.2%) classified into PspA family 3 (PspA3). Four isolates (1.6%) were classified as PspA NT. Three isolates (1.2%) was identified as a PspA null strain.

**Figure 1:**
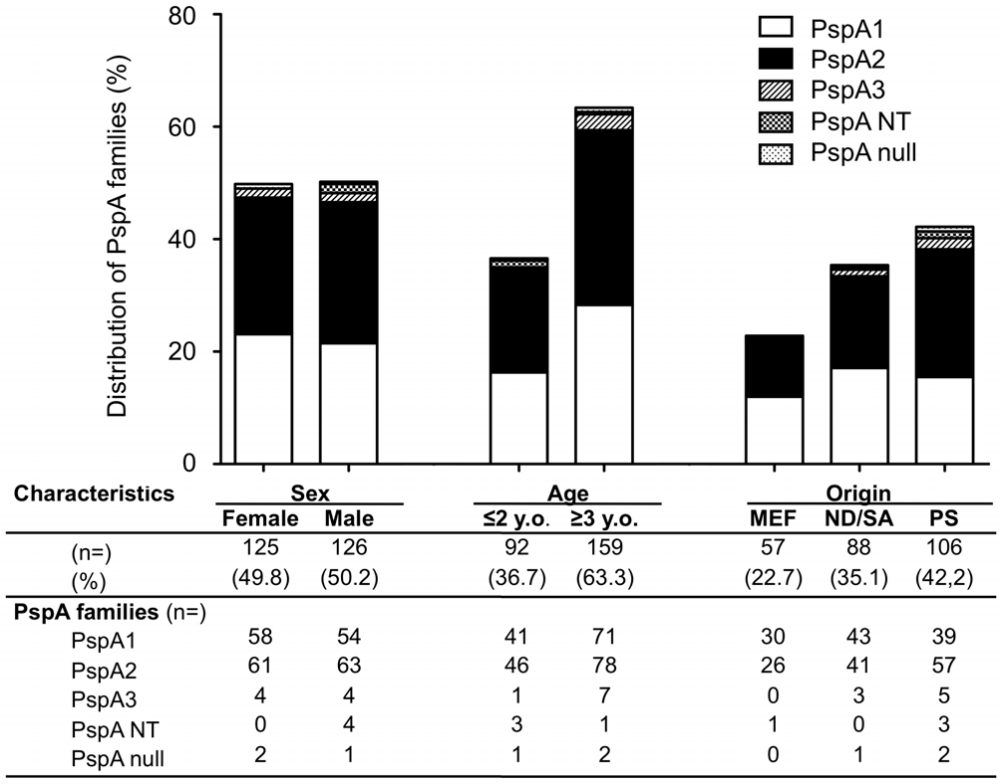
Distribution of PspA familes based on sex, age and origin of pneumococci. MEF: middle ear fluid, ND/SA: nasal discharge/sinus aspirate, PS: pharyngeal secretion. Each numbers shows numbers of isolates and percentage shows in parenthesis. There is no significant differences in PspA family distribution based on sex, age and origin of isolates.

**Figure 2:**
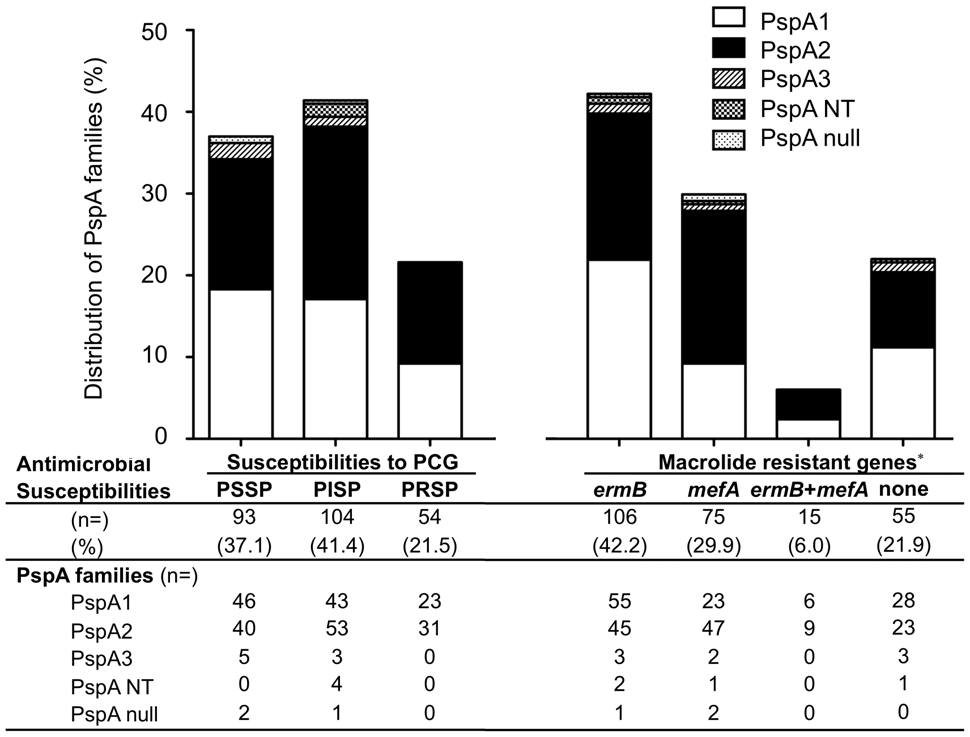
Distribution of PspA familes based on antimicoribila suscptibilities. PSSP: penicillin susceptible *S. pneumoniae*, PISP: Penicillin intermediately resistant *S. pneumoniae*, PRSP: penicillin resistant *S. pneumoniae*. Each numbers shows numbers of isolates and percentage shows in parenthesis. *p<0.05.

Because the vast majority of PspA families were identified as PspA1 or PspA2, we further evaluated the distributions of PspA1 and PspA2 by the other parameters. There were no significant differences in the distributions of PspA1 and PspA2 based on the age and gender of the patients, the origin of the isolates (Fig. 1.). Although there were no significant differences in the distribution of PspA1 and PspA2 based on the isolates’ susceptibilities to PCG, PspA2 were expressed at a higher frequency among the strains with the *mefA* gene (OR 2.4, 95% CI 1.4–4.1, *p* = 0.003) than the population of strains in general (Fig. 2.).

**Figure 3:**
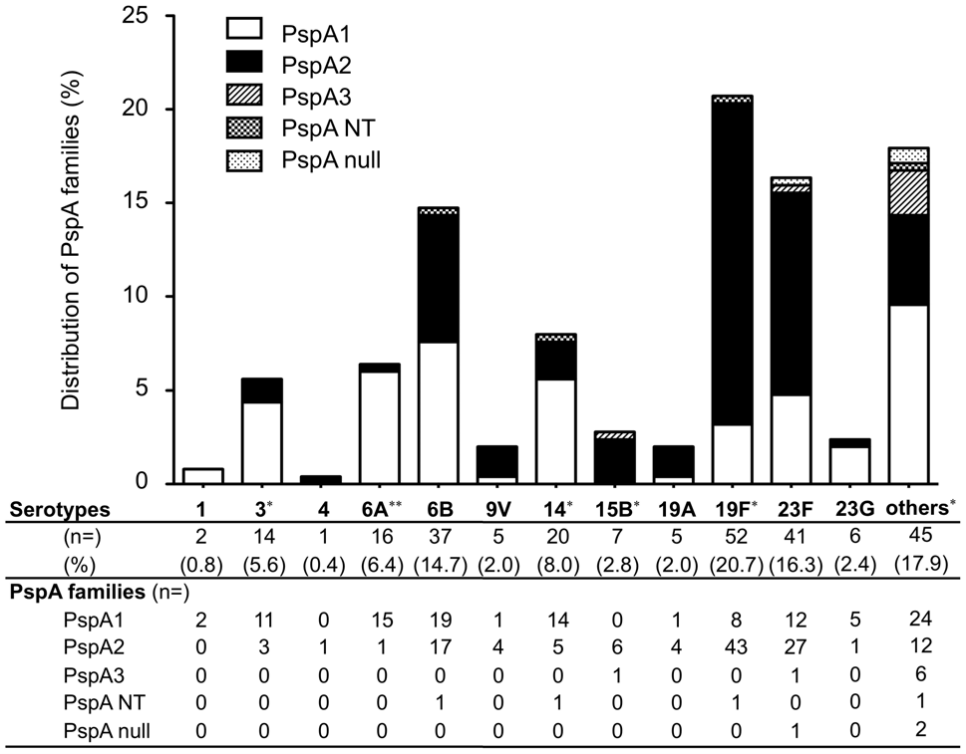
Distribution of PspA familes based on pneumococcal serotypes. G23: serogroup 23 strains except serotype 23F. Others: serotypes not included in 23 PPV. Each numbers shows numbers of isolates and percentage shows in parenthesis. *p<0.05, **p<0.01.

The distribution of PspA families based on their serotypes is shown in Fig. 3. The differences in distribution of PspA1 and PspA2 isolates based on their pneumococcal serotype were statistically significant (*p* = 0.013). Serotype 19F (OR 6.9, 95% CI 3.1–15.5, *p*<0.001), and serotype 15B (OR 12.3, 95% CI 0.7–221.8, *p* = 0.031) frequently expressed PspA2. Serotype 3 (OR 4.4, 95% CI 1.2–16.2, *p* = 0.025), serotype 6A (OR 19.0, 95% CI 1.7–146.6, *p*<0.001) and serotype 14 (OR 3.4, 95% CI 1.2–9.8, *p* = 0.029) tended to express PspA1. Serotypes 6B contained equal numbers of PspA1 and PspA2 isolates. In spite of these statistical differences in PspA family frequency among the different capsular types, representative capsular types (except for serotype 15B) had isolates of both PspA families. Thus, in general the capsular types were found not to be restricted to particular PspA families and PspA families were not restricted to particular capsular types.

### Coverage of Pneumococcal Vaccine Formulas

The coverage and 95% CI of pneumococcal vaccine formulas according to serotypes and PspA families are listed in Table 3. The total serotype coverage of the 7-valent, 10-valent (10 PCV), 13-valent (13 PCV), and 23-valent pneumococcal vaccines were 62.2%, 62.9%, 76.9% and 70.5%, respectively. The coverage of DRSP by the 7-valent, 10-valent, 13-valent, and 23-valent pneumococcal vaccines were 74.7%, 75.3%, 82.9% and 78.5%, respectively. The total coverage of *S. pneumoniae* with either the *mefA* gene or the *ermB* gene by the 7-valent, 10-valent, 13-valent, and 23-valent pneumococcal vaccines were 68.4%, 68.9%, 81.1%, and 74.5%, respectively. The percentages of total pneumococcal isolates, DRSP, and macrolide resistant *S. pneumoniae* (MRSP) having either *mefA* or *ermB* gene strains that would be covered by a PspA vaccine including the PspA1 and PspA2 families were 94.0%, 94.9%, and 94.4%, respectively. Consequently, the serotype coverage by a PspA vaccine was higher than the serotype coverage provided by the current 7 PCV, 10 PCV, 13 PCV, and 23 PPV vaccines (*p*<0.001).

**Table 3:** Serotype coverage of pneumococcal vaccine formulas among *S. pneumoniae* isolates from upper respiratory tract infections in Japan.

Vaccine formulations	Number and percentable coverage of *S. pneumoniae*					
	DRSP (n = 158)		MRSP (n = 196)		Total (n = 251)	
	n (%)	95% CI	n (%)	95% CI	n (%)	95% CI
7-valent (4,6B,9V,14,18C,19F,23F)	118 (74.7%)	67.9%−81.5%	134 (68.4%)	61.9%−74.9%	156 (62.2%)	56.2%−68.2%
10-valent (1,4,5,6B,7F,9V,14,18C,19F,23F)	119 (75.3%)	68.6%−82.0%	135 (68.9%)	62.4%−75.4%	158 (62.9%)	57.0%−68.9%
13-valent (1,3,4,5,6A,6B,7F,9V,14,18C,19A,19F,23F)	131 (82.9%)	77.0%−88.8%	159 (81.1%)	75.6%−86.6%	193 (76.9%)	71.7%−82.1%
23-valent (1,2,3,4,5,6B,7F,8,9V,9N,10A,11A,12F,14,15B,17F,18C,19A,19F,20,22F,23F,33F)	124 (78.5%)	72.1%−84.9%	146 (74.5%)	68.4%−80.6%	177 (70.5%)	64.9%−76.2%
PspA (PspA1 and PspA2)	150 (94.9%)	91.5%−98.4%	185 (94.4%)	91.2%−97.6%	236 (94.0%)	91.1%−97.0%

DRSP: drug resistant *S. pneumoniae* (PISP+PRSP). MRSP: macrolide resistant *S. pneumoniae*.

## Discussion

PspA consists of five domains including a signal peptide, alpha-helical charged region, a proline-rich domain, a choline-binding domain consisting of ten amino acids repeats, and a C-terminal amino acid tail [37]–[39]. Depending on the divergence of nucleotide sequences in the alpha-helical charged region, PspA is classified into three families, with no more than 50% sequence divergence within each family. The three PspA families are made up of six PspA clades that diverge from each other by no more than 20% sequence identity within each clade; family 1 (clades 1 and 2), family 2 (clades 3, 4, and 5), and family 3 (clade 6) [38], [40], [41].

Despite the great variation in the sequences of PspA, mouse and humans antibodies against PspA can be cross-reactive and cross protective against invasive disease in mice [27], [28]. The serologic cross-reactivity of PspA has been found to be strongly associated with PspA, but not restricted to family [42], [43]. Even so these antibodies can be more cross-protective than their level of cross-reactivity might suggest. Immunization of adult humans and mice with a PspA family 1 produced antibodies that could protect mice from infection with strains of PspA families 1 or 2 and from infections with strains of 3 different capsular types [42], [44], [45]. In addition successful fusion proteins have been made between family 1 and family 2 PspAs that can elicit antibody in mice protect against challenge strains of both PspA families [46]. In this study we focused on the distribution of PspA families among clinical isolates in Japan.

The Japanese strains were evenly distributed over family 1 and family 2. The proportions of the different PspA families can vary somewhat among countries. Hollingshead et al reported that the majority of PspAs in a collection of strains from Alabama fell into family 1 [37]. A study on invasive pneumococcal strains isolated from children less than 5 years of age in Colombia showed that 62.5% and 35.0% of strains belonged to families 1 and 2, respectively [43]. In Argentina 54.4% and 41.6% of the strains belonged to family 1 and family 2, respectively, with only 4.0% of the strains isolated from children being unclassifiable [47]. In Brazil, 50.5% of the isolates belonged to family 1, 43.2% were members of family 2, and 6.3% were not classified [48], [49]. In contrast, the high prevalence of PspA family 2 among pneumococci isolated from invasive pneumococcal diseases has been reported from Spain, Poland, Canada, Sweden, Germany, the USA, and France [37], [50]–[53]. A recent study of pneumococci isolates from nasopharyngeal carriage in Finnish children showed a prevalence of PspA family 1 and family 2 that was similar to our results [54]. The vast majority of pneumococci isolated from the middle ear fluid or nasopharyngeal secretion samples of the Finnish children less than 2 years old were from PspA families 1 and 2 [54]. Prior to our study, there had been a few reports of the PspA family distribution among pneumococci in Japan or any other countries in Asia [55], [56].

In contrast to the similar frequencies of PspA1 and PspA2 in Japan the frequency of different capsular serotypes was highly variable with 19F, 23F, 14, 6A, 6B, and 3 being the predominant common capsular types we observed which together accounted for 71.7% of the pneumococci isolates in this study. However, the PspA family distribution varied somewhat among serotypes. Earlier studies found that both PspA families occurred within the most common capsular serotypes, but that some serotypes were associated more strongly with one PspA family than the other [57], [58]. The capsular serotypes most strongly associated with a certain PspA family are 9N, 9V, 11A, 14, and 23F, whereas serotypes 6A, 6B, 19A, and 19F were equally associated with PspA families 1 and 2. This was most dramatic for the 24 different 23F isolates which were 25% PspA1 and 75% PspA2. In a study in France 37 different 23F strains were examined; 92% were PspA1 and 8% were PspA2 [54]. These findings indicate that there can be variations of distributions in PspAs in different geographic different regions although serotypes do not necessarily globally associate with certain PspAs. In some regions some capsular serotypes associated with a certain PspA family might be heavily clonal.

Based on the previously published information on PspA family distribution, there is still little information about the relationship between PspA families and antimicrobial-susceptibilities. In Japan, the rate of antimicrobial-resistant *S. pneumoniae* has increased continually since around 1990 and was about 49.0% between 1998 and 2000 [59], [60]. As documented in previous reports, penicillin-resistant strains were frequently identified among children younger than 2 years old [61]. In our previous study most of the serotype 19F and 23F strains were classified as either PISP or PRSP, while all of serotype 3 stains were classified as PSSP in middle ear isolates [62]. In this study, PRSP strains consisted equally of family 1 and 2 PspA. This means that a PspA-based vaccine would show a higher coverage of PRSP compared to the polysaccharide-based vaccines that have been available in the market.

Previous studies showed that PspA clades were independent of capsular serotypes [49], [50], [51]. Pneumococci of the same serotype were associated with different PspA clades from the same or a different family. This means that PspA-containing vaccines may be able to improve the protective efficacy of pneumococcal vaccines compared with the currently available serotype-based vaccines and may be able to avoid the serotype replacement that has been observed with conjugate vaccines [16]. The coverage of serotypes and PRSP by the 7 PCV was reported to be 62.8% and 88.0% for middle ear isolates, respectively. A PspA-based vaccine that contained representatives of PspA families 1 and 2 would potentially provide a high coverage rate because it would be cross-protective against invasive disease caused by the bulk of pneumococci infecting children and adults. It will be important however that data relating to both serotype and antibiotic resistance, similar to those reported here for Japan, should be collected in other geographical areas. Such a study would help to determine if a vaccine covering PspA families 1 and 2 would be appropriate for the geographic region in question.

In conclusion, even conjugate vaccine formulations with 13 pneumococcal capsular polysaccharides will not reach the coverage of 90% or more achieved by a vaccine containing family 1 and 2 PspA. The addition of PspA to the existing conjugate vaccine formulations may be a possible alternative for future development of pneumococcal vaccine.
